# Amelioration of obsessive-compulsive disorder by intracellular acidification of cortical neurons with a proton pump inhibitor

**DOI:** 10.1038/s41398-024-02731-3

**Published:** 2024-01-16

**Authors:** Hikari Hatakama, Nozomi Asaoka, Kazuki Nagayasu, Hisashi Shirakawa, Shuji Kaneko

**Affiliations:** 1 Department of Molecular Pharmacology, Graduate School of Pharmaceutical Sciences, Kyoto University, 46-29 Yoshida-Shimoadachi-cho, Sakyo-ku, Kyoto, 606-8501 Japan; 2 Department of Pharmacology, Graduate School of Medicine, Kyoto University, Yoshida-Konoe-cho, Sakyo-ku, Kyoto, 606-8501 Japan

**Keywords:** Psychiatric disorders, Pharmacology

## Abstract

Obsessive-compulsive disorder (OCD) is a highly prevalent neuropsychiatric disorder poorly controlled with pharmacological treatment because of the wide variation in symptom patterns. We analysed real-world data on adverse self-reports and insurance claims to identify a novel therapeutic target for OCD. We found that dopamine D_2_ receptor (D_2_R) agonists increased the incidence of OCD-like symptoms, which were suppressed by the concomitant use of proton pump inhibitors (PPIs). Further, OCD-like repetitive and habitual behaviours were observed in mice repeatedly injected with a D_2_R agonist, quinpirole. However, these abnormalities were suppressed by short-term PPI treatment. In quinpirole-treated mice, PPI inhibited pyramidal neuron hyperactivity in the lateral orbitofrontal cortex, a region where the P-type proton pump gene *Atp4a* is abundantly expressed. In primary cultured cortical neurons, short-term PPI treatment lowered intracellular pH and decreased firing activity, which was mimicked by *Atp4a* knockdown. Our findings show that inhibition of P-type proton pumps may be a novel therapeutic strategy for OCD.

## Introduction

Excessive repetitive, intrusive urges (obsession) and inadequate behaviours (compulsion) are the major symptoms of obsessive-compulsive disorder (OCD) [1]. Selective serotonin reuptake inhibitors (SSRIs) are the first-choice treatment for OCD [2] but are limited by the late onset of therapeutic effects and high dosage requirements [2]. Additionally, 40–60% of patients with OCD do not respond to SSRI treatment even after a sufficient treatment duration [2, 3]. Accordingly, there is an urgent need for rapid-acting and effective therapeutic strategies for OCD.

In translational studies, differences between humans and animals impede the prediction of clinical efficacy from animal experiments [4]. Accordingly, we previously developed a reverse translational approach by combining incidence and retrospective analysis of clinical big data [5]. Calculating the incident rates allows analysis of the correlation of the occurrence of clinical symptoms with the use of a particular drug. Therefore, it is possible to comprehensively assess the ability of clinically used agents to lower the occurrence of adverse events (i.e. drug-repurposing potentials) [6–8].

Drug reactions can cause obsession and compulsion, with OCD-like symptoms observed in patients taking dopaminergic agents [9, 10]. As dopaminergic abnormalities (e.g. a decrease in the binding availability of striatal D_2_R [11]) have been reported in patients with primary OCD, there may be pathological similarities between patients with primary OCD and medication-induced OCD-like symptoms, especially dopaminergic abnormalities. Consistent with this postulation, we found that repeated administration of a D_2_R agonist (quinpirole; QNP) induced OCD-like repetitive and perseverative behaviours in mice [12, 13]. This finding demonstrates a common therapeutic strategy for primary OCD and medication-induced OCD-like symptoms.

This study elucidated therapeutic agents for OCD using a reverse translational analytical approach to two clinical datasets: the FDA Adverse Event Reporting System (FAERS) data and insurance claims in the USA obtained from IBM^®^ Watson Health^®^ (MarketScan data). After the data-driven identification of candidate therapeutic agents, we preclinically evaluated the effects of the drugs on mice exhibiting OCD-like behaviours as a proof of concept.

## Materials and methods

### Analysis of FAERS data

We analysed big clinical data as previously reported [6–8]. FAERS adverse event reports from the first quarter of 2004 to the fourth quarter of 2019 were downloaded from the FDA website. We analysed 11,438,031 remaining cases after eliminating duplicate reports. The search terms for OCD-like symptoms were selected from the narrow scope of the preferred term (PT, MedDRA version 23.0; Supplementary Table 1). Arbitrary drug names, including trade names and abbreviations, were mapped into unified generic names using the Medical Subject Headings descriptor (MeSH) ID (Supplementary Tables 2 and 3). Adverse event risk was evaluated by calculating the ROR using the *Z* score as reported previously [6–8]. A significant association between the drug of interest and OCD-like symptoms was indicated by a *Z* score >1.96.

### Analysis of MarketScan data

Insurance claims data from January 2017 to December 2019 were purchased from IBM^®^ Watson Health^®^ (Armonk, NY). The dataset of Medicare and Medicaid programmes in the United States contains the medical diagnoses and prescription claims of 43,723,094 anonymised employees and their dependents daily. Individual diagnoses were assigned based on the International Classification of disease 10 (ICD-10). Supplementary Tables 4 and 5 show the number of patients who used D_2_R agonists or PPIs. Cases of OCD-like symptoms were identified using the ICD-10 (Supplementary Table 6).

**Fig. 1:**
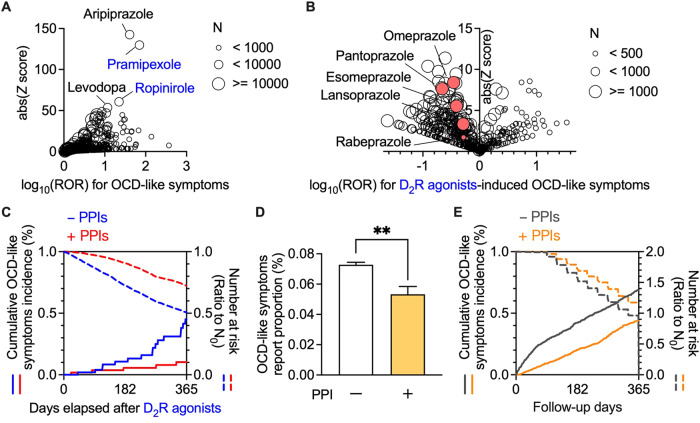
Analyses of FAERS data and MarketScan data revealed that concomitant use of proton pump inhibitors suppressed the incidence of OCD-like symptoms. **A**, **B** In FAERS data, the volcano plot indicates drug interactions with OCD-like symptoms using the reporting odds ratio (ROR, log scale) and statistical significance (absolute *Z* score). Each plotted point represents an individual drug, and the plot size reflects the number of patients taking the drug. There was a significant increase in the ROR of OCD-like symptoms in patients using D_2_R agonists, including pramipexole and ropinirole (**A**). Among D_2_R agonist users, the confounding effects of concomitantly used drugs on the ROR of drug-induced OCD-like symptoms are plotted (**B**). **C** Occurrence of OCD-like symptoms within one year of D_2_R agonists administration. In MarketScan data, Kaplan–Meier curves for the cumulative incidence ratio of OCD-like symptoms in D_2_R agonists users are shown in each of the two populations [one without (blue) and one with (red) prescription of proton pump inhibitors (PPIs)]. The dotted lines show the number of patients at risk as a ratio to the initial number (*N*_0_) of patients. **D** Effects of PPIs on the report proportion of OCD-like symptoms in non-D_2_R agonists users in FARES data. **P < 0.01. **E** Occurrence of OCD-like symptoms within one year from 90 days after enrolment. In MarketScan data, Kaplan–Meier curves for the cumulative incidence ratio of OCD-like symptoms in non-D_2_R agonists users are shown in each of the two populations [one without (grey) and one with (orange) prescription of PPIs]. The dotted lines show the number of patients at risk as a ratio to the initial number (*N*_0_) of patients.

We analysed the confounding factors in the propensity score matching (Supplementary Table 7). Analyses of MarketScan data were performed using R. Further, time series analyses were performed using the R packages ‘survival’ (version 3.2.3) and ‘MatchIt’ (version 3.0.2). For Fig. 1E, samples were randomly extracted using the sample_n() function of R packages ‘dplyr’ (version 1.1.1).

After each cohort was divided into two groups (with and without PPIs), we used 1:1 propensity score matching [14] to eliminate deflections in the number of patients with risk factors by matching two groups using the nearest-neighbour method with a 0.01 (Fig. 1D) or 0.2 (Fig. 1E) calliper width [15]. The chi-square and Fisher’s exact tests were used to compare population characteristics. The cumulative incidences of OCD-like symptoms were compared between patients with and without concomitant use of PPIs through conventional survival analysis [16]. The number at risk indicates the number of patients who may have an onset of OCD-like symptoms each day. Statistical significance was evaluated using the log-rank test and Cox proportional regression to calculate the hazard ratio.

Using the matched cohort pairs, the daily doses, cumulative doses, and administration periods of D_2_R agonists, levodopa, and PPIs were quantified and compared using an unpaired *t* test with Welch’s correction.

### Animals

All animal care and experimental procedures were conducted following the ethical guidelines of the Kyoto University Animal Research Committee. Male C57BL/6J mice were purchased from Nihon SLC (Shizuoka, Japan). Mice (6–15 weeks old) were housed at a constant ambient temperature of 24 ± 1 °C on a 12 h light–dark cycle with ad libitum access to food and water. As previously reported [12, 17], the mice were intraperitoneally injected with QNP (1 mg/kg) weekday; citalopram hydrobromide was dissolved in drinking water (0.2 mg/mL) and administered for 4 weeks.

### Recording of repetitive behaviour

The mice were singly housed; subsequently, spontaneous behaviour in their home cage was recorded starting from 20 min after the last drug injection for 10 min, when chewing was most strongly induced, as previously defined [12, 13]. Chewing was defined as holding a wood chip in the forelimbs and biting and pulling it with the mouth and forelimbs. Walking was defined as taking a step forward without chewing. Data analysis was performed in a blinded manner.

### Operant training

Before training commenced, the mice were food-restricted (2–2.2 g/day; 80–90% of the ad libitum-fed body weight). Next, the mice were placed in an operant chamber with a house light, a single lever, and a single reward delivery port in a sound-attenuating box. Each component of the operant chamber (Med Associates, St Albans, VT) was controlled using the MED-PC IV software (Med Associates). The training session began with the extension of the lever and turning on the house light. It ended with retracting the lever and turning off the house light. The training was performed for 11 days [18]. For habituation, the mice received an average of 15 reinforcers (10 μL of 20% solution per reinforcer) at 60-s intervals without lever presentation in the operant chamber for 2 days. Each lever press was rewarded on a 3-day continuous reinforcement schedule; the number of available reinforcers increased with each training day (5, 15, and 30). Next, the mice underwent a 6-day RR reinforcement schedule. Subsequently, the mice were trained on the RR10 schedule (10 lever presses on average for one outcome) for 2 days and the RR20 schedule (20 lever presses on average for one outcome) for 4 days. Each RR training session was terminated after the mice had received 15 reinforcers or 60 min had elapsed.

After the 6-day RR training sessions, the outcome devaluation test was conducted over two consecutive days. In the devaluation test, the mice were allowed free access to food pellets (valued day) or sucrose solution (devalued day) for 30 min. Immediately after the free access session, the mice completed a 5-min lever pressing session where the lever was presented as usual, but the reward was never delivered. The devaluation index was determined as follows: [(lever presses on the valued day) − (lever presses on the devalued day)/[(lever presses on the valued day) + (lever presses on the devalued day)]. The order of the valued and devalued days was randomised.

### Preparation of brain tissue samples

As previously described [13], each brain region of 1-mm coronal brain slices was removed based on the Brain Atlas [19].

### Preparation and delivery of AAV vectors

To construct AAV-hSyn-mCherry-SEpHluorin-miRNA, the hSyn-mCherry-SEpHluorin fragment was amplified from hSyn-mCherry-SEpHluorin (#32001; Addgene Plasmid) using PCR and ligated to the AAV backbone obtained from pAAV-CMV-EmGFP-miRNA [20]. The miRNA targeting sequence used is shown in the Supplementary Methods.

### Electrophysiological recordings

Electrophysiological recordings were performed as previously described [17] with minor modifications; details of the brain preparation are provided in the Supplementary Methods.

A coverslip was transferred to a recording chamber filled with oxygenated ACSF to record cultured cortical neurons, and recordings were performed at room temperature. The detailed method of primary cortex neuronal culture is shown in the Supplementary Methods.

Electrophysiological recordings were performed with an EPC9 amplifier (HEKA, Pfalz, Germany) and recorded using Patchmaster software (HEKA). The resistance of the electrodes was 3–7 MΩ when filled with the intracellular solution (140 mM K-gluconate, 5 mM KCl, 10 mM HEPES, 2 mM Na-ATP, 2 mM MgCl_2_, and 0.2 mM EGTA; pH 7.3 or 7.0, adjusted with KOH). The concentration of HEPES was selected following the previous report demonstrating intracellular acidification induced by current injection [21]. Individual neurons were visualised using a microscope with a ×40 water-immersion objective lens (Zeiss, Jena, Germany) and a CCD camera. The series resistance was compensated by 70% and maintained within 35 MΩ. Pyramidal neurons were defined by regular spiking responses to the current injection (300 pA, 1 s duration) [12].

Neural excitability recordings were obtained under current clamp conditions (holding current = 0 pA). Action potentials were evoked using a current injection (0–500 pA, 1 s duration). In the experiments with the vonoprazan application, firing responses were recorded before and after the 5-min vonoprazan bath (10 μM). Since almost all AAV-infected cultured neurons stopped firing after injection of >400 pA current, we analysed the 0–300 pA conditions for the KD experiments.

### Measurement of intracellular pH

Cells on coverslips were placed in Krebs–Ringer solution (140 mM NaCl, 3 mM KCl, 1 mM MgCl_2_, 2 mM CaCl_2_, 10 mM glucose, and 10 mM HEPES; pH 7.3, adjusted with NaOH); SEpHluorin-derived fluorescence images were captured at 1-s intervals using alternating excitation at 480 nm with an AQUACOSMOS/ORCA-AG imaging system (Hamamatsu Photonics, Shizuoka, Japan). For calibration, neurons were incubated in a high K^+^ Krebs–Ringer solution (3 mM NaCl, 140 mM KCl, 1 mM MgCl_2_, 2 mM CaCl_2_, 10 mM glucose, 10 mM HEPES, and 5 μM nigericin; pH 6.7–7.9, adjusted with NaOH).　After vonoprazan application, decreasing firing activity induced intracellular alkalinisation [22]; the effect on neuronal activity was abolished by pretreatment with tetrodotoxin (0.3 μM) 15 min before vonoprazan application. In the analysis, baseline SEpHluorin fluorescence intensity was normalised to the average of a 2-min period at 1 min before vonoprazan application; moreover, GFP fluorescence intensity during vonoprazan application was calculated 5 min after the application. For *Atp4a* KD cells, GFP fluorescence intensity was normalised to the average of a 2-min period within 9 min after measurement.

### Statistical analysis

The sample size was determined based on previous reports performing similar experiments, or the minimum sample size required was calculated using R packages (‘pwr’ version 1.3-0, ‘powerSurvEpi’ version 0.1.3) or G*Power software version 3.1.9.7. All in vivo and in vitro data are presented as mean ± S.E.M. Statistical analyses were performed using GraphPad Prism, version 9.3.1. Statistical significance was set at *P* < 0.05. The detailed statistical method is shown in the Supplementary Methods.

## Results

### Concomitant use of PPIs suppressed the incidence of OCD-like symptoms induced by D_2_R agonists

We first analysed FAERS data to investigate the association between drug use and OCD-like symptoms (Supplementary Table 1). A disproportionality analysis revealed a significant association between the use of dopamine receptor stimulants and increased occurrence of OCD-like symptoms (Fig. 1A and Supplementary Table 2). Among the dopamine receptor stimulants, pramipexole, and ropinirole, which are D_2_R agonists, showed high reporting odds ratios (ROR) and *Z* scores. Moreover, aripiprazole, widely used as an augmentation therapy for patients with treatment-resistant OCD, was strongly associated with the emergence of OCD-like symptoms [23]. We observed a relationship between the use of D_2_R agonists and OCD-like symptoms through a comprehensive analysis of FAERS data.

Next, we evaluated the confounding effects of all drug combinations among D_2_R agonists users (Fig. 1B and Supplementary Table 3). Among the concomitant drugs which decreased the incidence of OCD-like symptoms induced by D_2_R agonists, a class effect was observed in the following three drug groups: vitamin D (cholecalciferol and ergocalciferol), loop diuretics (furosemide, torsemide, and bumetanide), and proton pump inhibitors (PPIs; pantoprazole, omeprazole, esomeprazole, lansoprazole, and rabeprazole). Vitamin D was excluded, given its therapeutic effect on restless legs syndrome, which is frequently treated with D_2_R agonists [24], as it may reduce the dosage of D_2_R agonists. Furthermore, loop diuretics were omitted since renal dysfunction can affect the pharmacokinetics of pramipexole, which is mainly excreted in the urine [25]. Accordingly, we focused on PPIs as a potential concomitant drug inhibiting OCD-like symptoms.

We subsequently analysed the effects of the use of PPIs and the onset of OCD-like symptoms induced by D_2_R agonists to overcome the reporting bias and lack of a denominator for incidence because of self-reporting. This analysis used MarketScan data, which contains information on the number of D_2_R agonists users and their prescription dates (Supplementary Fig. 1A, B and Supplementary Tables 4–6).

Patients who received PPIs were defined as those prescribed PPIs for the first time after using D_2_R agonists. We performed 1:1 propensity score matching (Supplementary Tables 7–11) to eliminate known confounding factors [2, 26–28].

We analysed the daily doses, cumulative doses, and administration periods of D_2_R agonists, levodopa, and PPIs to validate the matched cohorts (Table 1). The doses of D_2_R agonists and PPIs were converted to the levodopa equivalent dose (LED) [29] and omeprazole equivalent dose (OED) [30], respectively. The daily doses of D_2_R agonists and PPIs were within the standard dose range in the USA (D_2_R agonists: LED 12.5–450, PPIs: OED 5–40).

**Table 1:** Daily and cumulative doses and administration periods of D_2_R agonists, levodopa, and proton pump inhibitors (PPIs) in the propensity score-matched cohorts when used in combination with proton pump inhibitors of MarketScan data using l-dopa equivalent dose (LED) and omeprazole equivalent dose (OED).

		Without PPIs (*n* = 5528)	With PPIs (*n* = 5528)	*P* value	*T* value
D_2_R agonists	Average daily dose (LED, median and IQR)	20.0 (10.0–50.0)	25.0 (12.5–50.0)	0.123	*t*(10,617) = 1.54
	Cumulative dose (LED, median and IQR)	2250 (750–8834)	4200 (1125–15,900)	0.503	*t*(8613) = 0.667
	Administration period (day, median and IQR)	90 (30–300)	180 (60–450)	<0.001	*t*(10,541) = 15.0
Levodopa	Average daily dose (LED, median and IQR)	347 (229–577)	350 (290–7645)	0.887	*t*(489) = 0.143
	Cumulative dose (LED, median and IQR)	117,000 (24,000–344,250)	156,000 (36,000–421,650)	0.501	*t*(450) = 0.674
	Administration period (day, median and IQR)	330 (90–720)	360 (90–750)	0.398	*t*(449) = 0.847
PPIs	Average daily dose (OED, median and IQR)		20.1 (20.0–40.0)		
	Cumulative dose (OED, median and IQR)		3000 (1200–7200)		
	Administration period (day, median and IQR)		90 (30–240)		

Bias toward the levodopa doses may have affected the analysis because levodopa use is associated with OCD-like symptoms [31]. In both cohorts, the levodopa dose was reasonable (standard dose: LED 42–500), with no between-group difference in dose.

We performed a chronological sequence analysis to assess the inhibitory effects of PPIs on OCD-like symptoms induced by D_2_R agonists. Consistent with the FAERS analysis, Kaplan-Meier analysis, and Cox proportional hazards modelling indicated that the concomitant use of PPI(s) significantly decreased the incidence of OCD-like symptoms induced by D_2_R agonists, with a hazard ratio of 0.432 (Fig. 1C, 95% CI: 0.238–0.783, *P* = 0.004 in the log-rank test).

Concomitant use of H_2_ blocker(s) did not significantly affect the incidence of OCD-like symptoms induced by D_2_R agonists (Supplementary Fig. 1C, a hazard ratio of 1.10, 95% CI: 0.359–3.39, *P* = 0.090 in the log-rank test; Supplementary Tables 12–17), suggesting that the inhibitory effects of PPIs on the incidence of OCD-like symptoms did not result from the inhibition of gastric acid secretion and history of gastrointestinal diseases.

Furthermore, we investigated whether the administration of PPIs was effective for OCD-like symptoms in patients without D_2_R agonists. Analysis of FAERS data revealed that the use of PPIs significantly reduced the incidence of OCD-like symptoms in non-D_2_R agonists users (Fig. 1D). Additionally, in matched cohorts among non-D_2_R agonists users of MarketScan data, PPIs also significantly lowered the incidence of OCD-like symptoms with a hazard ratio of 0.533 (Fig. 1E, 95% CI: 0.483–0.589, *P* < 0.001 in the log-rank test; Supplementary Tables 18 and 19).

These findings showed that the concomitant use of PPIs alleviated OCD-like symptoms.

### PPI ameliorated behavioural repetition induced by repeated D_2_R stimulation

**Fig. 2:**
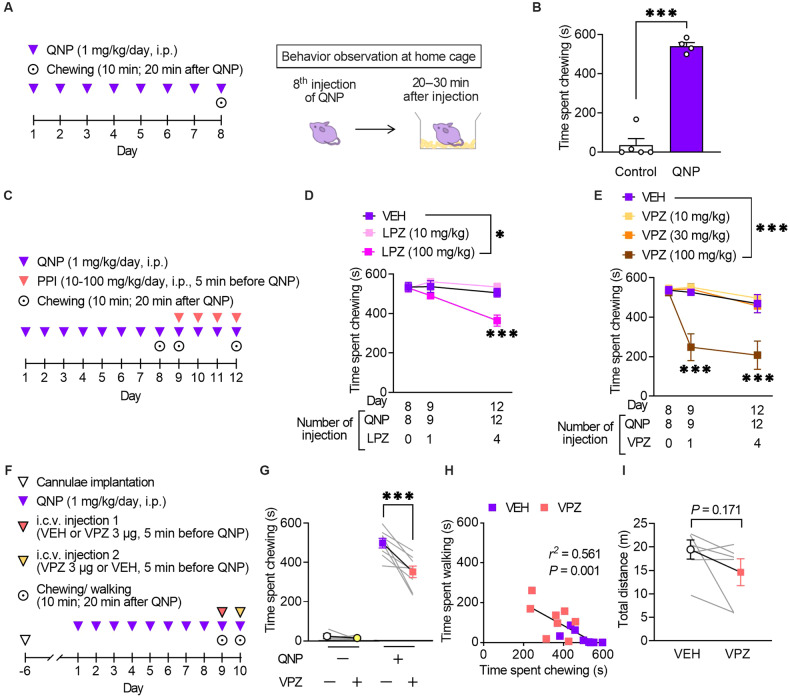
Vonoprazan administration improved repetitive behaviour in quinpirole-treated mice. **A** Mice received eight daily injections of quinpirole (QNP; D_2_R agonist; 1 mg/kg), and spontaneous behaviour was observed 20–30 min after the eighth injection of QNP. **B** The time spent chewing in the vehicle (control) or QNP-treated mice within 10 min. **C** Mice received intraperitoneal injections of a proton pump inhibitor (PPI) 5 min before the 9–12th QNP injection. Chewing was measured for 10 min starting from 20 min after QNP administration. **D** Time spent chewing in mice received lansoprazole (LPZ) for four days. **E** Time spent chewing in mice received vonoprazan (VPZ) for 4 days. **F** QNP injections were started seven days after cannula implantation into the cerebral ventricle. VEH or VPZ (3 μg) was injected through the cannula 5 min before the 9th or 10th QNP injection in a crossover design for the initial measurement of chewing and walking behaviour. **G** The effect of intracerebroventricular administration of VPZ (3 μg) on QNP-induced chewing behaviour. **H** Correlation between chewing and walking in mice treated with VEH or VPZ. *r*^2^ = 0.561, *P* = 0.001. **I** Effect of intracerebroventricular administration of VPZ (3 μg) on locomotor activity. *P < 0.05; ***P < 0.001.

Repeated injections of a D_2_R agonist (QNP) elicit OCD-like behavioural and neurological abnormalities in mice [12, 13, 17]. We assessed the effects of PPIs on QNP-induced OCD-like behaviours to obtain preclinical proof-of-concept. We previously showed that repeated injection of QNP induced repetitive behaviour [12, 13, 17]. Consistent with our previous observations, repeated intraperitoneal injection of QNP (1 mg/kg/day, eight times) significantly induced excessive repetition of spontaneous chewing behaviour (Fig. 2A, B). When mice were treated with lansoprazole (100 mg/kg) for four days before QNP administration (Fig. 2C), the time spent chewing was significantly decreased (Fig. 2D).

We validated the effects of vonoprazan, a potassium-competitive acid blocker with more rapid inhibition of P-type proton pumps (the main PPI targets) than conventional PPIs to identify the class effect of PPI [32]. Similarly, vonoprazan (100 mg/kg) inhibited the QNP-induced increase in repeated chewing behaviour (Fig. 2E). In contrast to lansoprazole, a single administration of vonoprazan exerted an inhibitory effect.

Mice received vonoprazan injections in the intracerebroventricular space to exclusively assess the effects of PPIs in the brain. Consistent with systemic vonoprazan administration, a single administration of vonoprazan (3 μg) ameliorated QNP-induced repeated chewing behaviour (Fig. 2F, G). Time spent chewing was negatively correlated with time spent walking (Fig. 2H). Intracerebroventricular injection of vonoprazan (3 μg) did not affect locomotor activity, demonstrating that the decreased chewing behaviour did not result from the sedative effect of vonoprazan (Fig. 2I).

### PPI ameliorated habitual action control induced by repeated D_2_R stimulation

Maladaptive habit-based decision-making and excessive/inappropriate repetition of actions are critical clinical characteristics of patients with OCD and OCD-like symptoms [1]. Recent animal studies have assessed OCD-like behavioural abnormalities through the facilitated formation of habitual behaviour [33, 34].

**Fig. 3:**
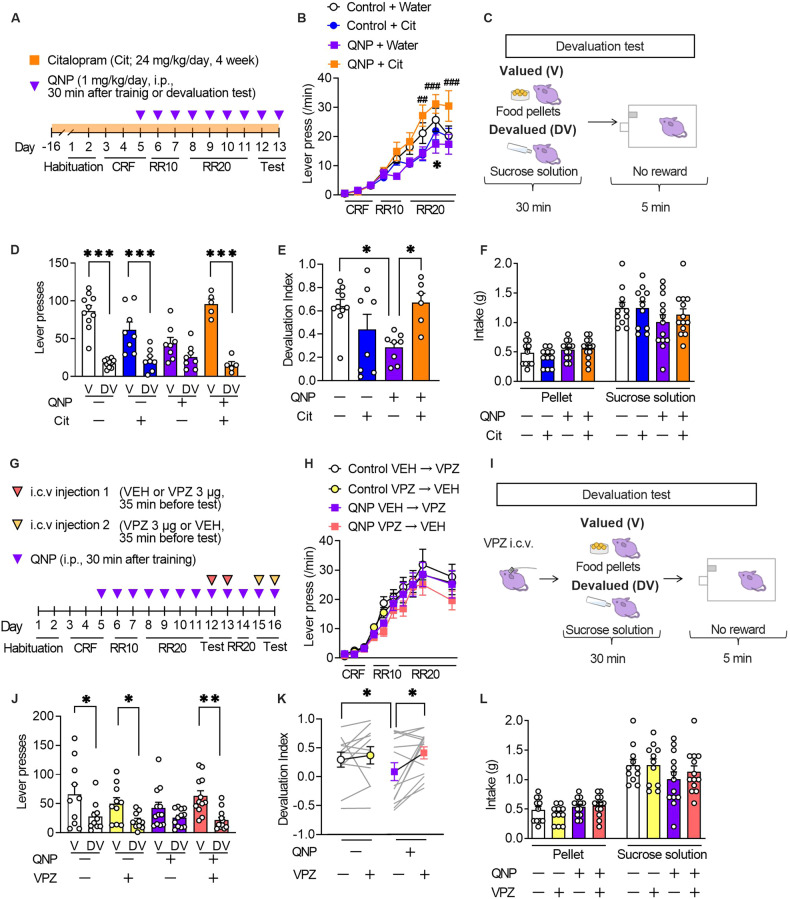
Vonoprazan administration improved habitual action in quinpirole-treated mice. **A** The mice underwent 11-day training on the random ratio (RR) schedule, followed by 2-day outcome devaluation testing. Mice were treated daily with QNP (1 mg/kg) for eight days; moreover, they orally received water or citalopram (24 mg/kg/day; Cit) for four weeks before the first outcome devaluation test. **B** Response speed of lever pressing during acquisition under operant training schedules. *P < 0.05 versus Control+Water groups, ^##^*P* < 0.01, ^###^*P* < 0.001 vs QNP+Water group. **C** In the devaluation test, mice were fed food pellets on the valued (V) day or sucrose solution on the devalued (DV) day for 30 min. After pre-feeding, a 5-min extinction test was performed. **D**, **E** The numbers of lever presses in the devaluation tests (**D**) and devaluation index (**E**). **F** Consumption of food pellets and sucrose solution during the 30-min free-feed period. **G** Mice underwent 11-day training on the random ratio (RR) schedule and were treated daily with QNP (1 mg/kg) for 8 days before the first outcome devaluation testing. Vehicle (VEH) or vonoprazan (VPZ; 3 µg) was injected for outcome devaluation testing on day 12 or 13. On day 14, the mice were retrained to press the lever. On day 15 or 16, the second set of outcome devaluation testing was performed in a crossover design. **H** The response rate of lever pressing during acquisition under operant training schedules. **I** On the days of devaluation tests, mice received an intracerebroventricular injection of VEH or VPZ (3 μg) 5 min before the free-feeding session. **J**, **K** The numbers of lever presses in the devaluation tests (**J**) and devaluation index (**K**). **L** Consumption of food pellets and sucrose solution in the mice during the 30-min free-feed period. *P < 0.05; **P < 0.01; ***P < 0.001.

The formation of habitual strategies in QNP-treated mice was assessed using the devaluation test following a self-paced operant conditioning task (Fig. 3A). We also assessed the effects of long-term administration of an SSRI, the first-choice treatment for OCD to evaluate the clinically relevant treatment response [2]. The 6-day training on the random ratio (RR) reinforcement schedule gradually increased the lever press rates in all treatment groups, which suggested successful learning. However, there were between-group differences in the late training phase (Fig. 3B).

The 6-day training on an RR schedule is widely used to develop goal-directed strategies for lever press [18]. In a goal-directed strategy, reduction in the reinforcer value by satiety (devaluation) decelerates lever presses in the subsequent 5-min unrewarded lever press session, with a lack of a decelerating effect suggesting the formation of a habitual strategy (Fig. 3C). Vehicle-treated control mice showed a significant reduction in the number of lever presses in the devalued condition, which suggested the successful formation of a goal-directed strategy. QNP-treated mice showed relatively similar lever pressing rates between the valued and devalued states compared with control mice. However, 4-week citalopram treatment altered the distribution of the number of lever presses in QNP-treated mice (Fig. 3D). Based on the devaluation index, 4-week administration of citalopram shifted goal-directed actions from QNP-induced habitual actions (Fig. 3E). There were no significant between-group differences in the consumption of both food pellets and sucrose solution during the free-feeding sessions (Fig. 3F). These results confirmed that repeated QNP injections did not result in goal-directed lever pressing; moreover, long-term SSRI treatment improved maladaptive habitual behaviour.

We then investigated whether PPI mitigates QNP-induced habitual behaviour (Fig. 3G). There was no significant between-group difference in the number of lever presses during training on an RR schedule (Fig. 3H). In the devaluation tests, vehicle or vonoprazan (3 μg) was injected 5 min before QNP injection. Further, the mice were fed food pellets on the valued day or sucrose solution on the devalued day freely in their home cage for 30 min. Each day, the mice underwent a 5-min lever pressing session immediately after the free access session. Lever presses on the valued or devalued day were recorded for the 2-day devaluation tests (Fig. 3I). In vehicle-injected QNP-treated mice, there was no statistically significant change in lever presses between valued or devalued condition. Contrastingly, vonoprazan injection restored the devaluation-induced reduction in lever presses (Fig. 3J). Consistently, vehicle-injected QNP-treated mice showed a significantly lower devaluation index than control mice, while vonoprazan increased the devaluation index in QNP-treated mice (Fig. 3K). In the free-feeding sessions, there was no significant between-group difference in the intake of pellets or sucrose solution (Fig. 3L). These results indicated that proton pump blockade could effectively ameliorate the QNP-induced habitual action.

### PPI attenuated hyperactivity in the lateral orbitofrontal cortex (OFC) pyramidal neurons induced by repeated D_2_R stimulation

**Fig. 4:**
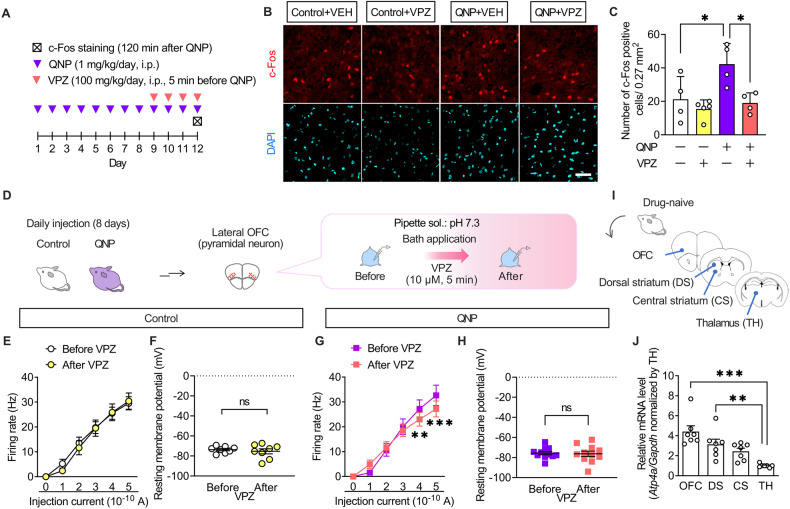
Administration of vonoprazan inhibited hyperactivity in lateral OFC pyramidal neurons obtained from QNP-treated mice. **A** Mice were intraperitoneally treated with QNP (1 mg/kg) for 8 days as well as the vehicle (VEH) or vonoprazan (VPZ; 100 mg/kg) 5 min before QNP injection on the 9th–12th day. Coronal sections containing the lateral OFC were prepared 120 min after the final QNP injection. **B** Representative images of c-Fos-positive cells in the lateral OFC. Red: c-Fos, a marker for activated neurons; Cyan: DAPI; Scale bar = 50 μm. **C** The number of c-Fos-positive cells in the lateral OFC. **D**–**H** The firing responses induced by current injection (0–500 pA; 1 s duration) were recorded before and after bath application of VPZ (10 μM) in intracellular pH 7.3 in the lateral OFC pyramidal neurons obtained from control (**E**, **F**) and QNP-treated mice (**G**, **H**). **E**, **G** Firing responses induced by current injection. **F**, **H** The resting membrane potential. **I** Samples containing brain regions of the cortico-striato-thalamo-cortical circuit were obtained from drug-naive mice. **J** Relative *Atp4a* mRNA levels in each brain region. ns: *P* > 0.05; *P < 0.05; **P < 0.01; ***P < 0.001.

The results of the intracerebroventricular injection experiments demonstrated that the effects of PPIs resulted from their action on the brain. Patients with OCD exhibit hyperactivity in the OFC ameliorated only in SSRI-responsive patients [35, 36]. Therefore, we hypothesised that PPIs modulate neural activity in the OFC. Accordingly, we examined the effects of vonoprazan (100 mg/kg) on the expression of c-Fos, a neural activity marker, in the lateral OFC through immunostaining (Fig. 4A). QNP-treated mice showed an increased number of c-Fos-positive cells in the lateral OFC. In contrast, vonoprazan injection decreased the number of c-Fos-positive cells in QNP-treated mice (Fig. 4B, C).

Further, we examined the effects of vonoprazan on the neural activity of lateral OFC pyramidal neurons using ex vivo patch-clamp recordings (Fig. 4D). Bath vonoprazan application (10 μM) did not affect the current-induced firing responses in lateral OFC pyramidal neurons compared with those in control mice (Fig. 4E, F). However, these were significantly decreased by vonoprazan treatment in QNP-treated mice (Fig. 4G, H). The inhibiting effects of vonoprazan on firing response were prominent in the late phase of the depolarising pulse (Supplementary Fig. 2).

The expression and function of P-type proton pumps in the brain remain unclear. We examined the expression pattern of *Atp4a* mRNA, which encodes the catalytic subunit of the P-type proton pump, to identify the brain regions involved in the effects of PPIs [37]. As patients with OCD have altered functional connectivity within the CSTC-loop [38, 39], we analysed mRNA expression levels of *Atp4a* in the CSTC-loop-related brain regions through real-time qRT-PCR (Fig. 4I). In drug-naive mice, *Atp4a* mRNA expression levels were higher in the OFC than in the thalamus (Fig. 4J). These results demonstrated that vonoprazan decreased neuronal hyperactivity in the lateral OFC of QNP-treated mice by inhibiting P-type proton pumps.

### PPI attenuated neuronal activity through intracellular acidification of primary cultured cortex neurons

**Fig. 5:**
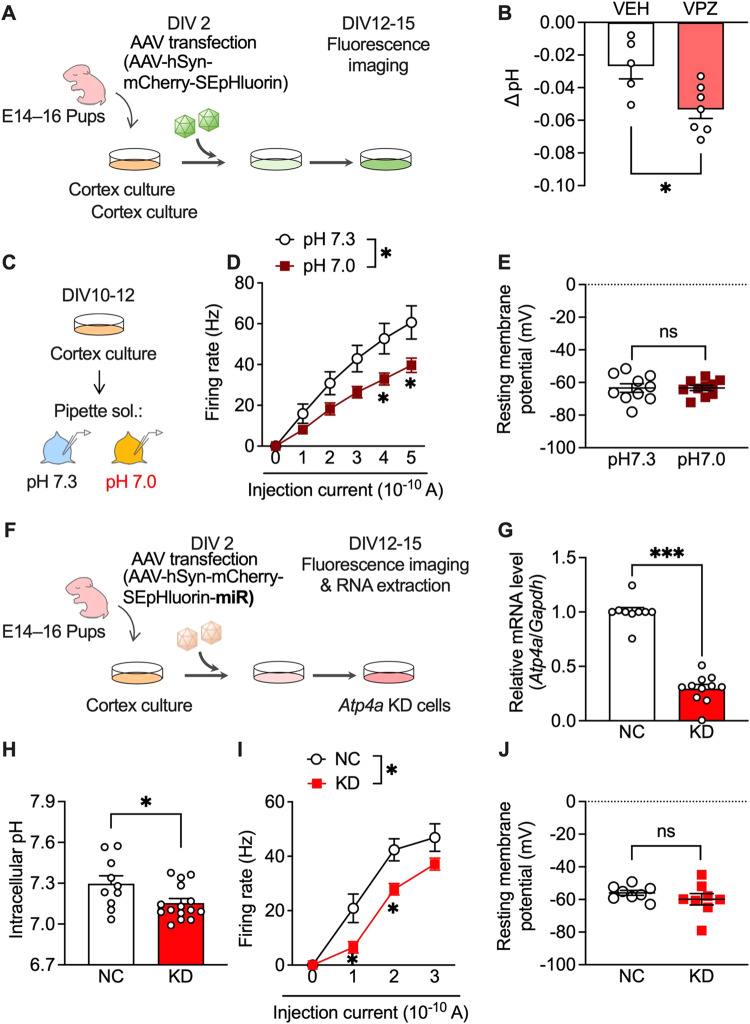
Administration of vonoprazan inhibited neuronal activity through intracellular acidification. **A** AAV-infected SEpHluorin-expressing primary cultures of mouse cortex neurons. **B** Intracellular pH at 5 min after vehicle (VEH) application or vonoprazan (VPZ; 10 μM). **C**–**E** Electrophysiological recordings of naive primary cultures of mouse cortex neurons in intracellular pH 7.3 or 7.0. **D** Firing responses induced by current injection (0–500 pA; 1 s duration). **E** The resting membrane potential. **F**–**J** AAV-SEpHluorin-miRNA-mediated negative control (NC) or *Atp4a* KD in primary cultures of mouse cortex neurons. **G** Relative expression levels of *Atp4a* mRNAs in cortex cultures at 12 days after AAV infection. **H** Intracellular pH of AAV-infected neuronal cultures. **I**, **J** Electrophysiological recordings of *Atp4a* KD primary cortex neurons. Firing activity elicited by current injection (**I**) and the resting membrane potential (**J**) is shown. ns: *P* > 0.05; *P < 0.05; ***P < 0.001.

Vonoprazan inhibits acid release into the extracellular environment through P-type proton pumps and may increase intracellular proton concentration [26]. Accordingly, we visualised the intracellular pH of primary cultured mouse cortical neurons using super-ecliptic pHluorin (SEpHluorin) [40–42], which is a pH-sensitive fluorescence protein that shows reduced fluorescence intensity under an acidic environment (Fig. 5A). Vonoprazan (10 μM) significantly decreased the intracellular pH compared with the vehicle (Fig. 5B).

Additionally, we examined the effects of intracellular acidification on neural activity in cultured cortical neurons. Firing responses induced by the depolarising current were recorded in normal (pH 7.3) and slightly acidic (pH 7.0) pipette solutions (Fig. 5C). The firing rate was significantly lower under acidic conditions than under normal conditions (Fig. 5D). There was no significant between-group difference in resting membrane potentials (Fig. 5E). These results showed that PPI-mediated intracellular acidification contributes to the inhibitory effects in lateral OFC neurons.

Next, we investigated whether P-type proton pumps in cortical neurons are crucial in controlling intracellular pH and neuronal activity. *Atp4a* knockdown (KD) was achieved using miRNA-encoding AAV (Fig. 5F), which significantly decreased *Atp4a* mRNA expression compared with AAV encoding the negative control (NC) miRNA (Fig. 5G). Consistent with vonoprazan experiment results (Fig. 5B), neuron-specific *Atp4a* KD significantly decreased the intracellular pH (Fig. 5H) and significantly inhibited the current-induced firing responses without altering the resting membrane potential (Fig. 5I, J).

### PPI attenuated neuronal activity under acidic intracellular conditions in lateral OFC pyramidal neurons

**Fig. 6:**
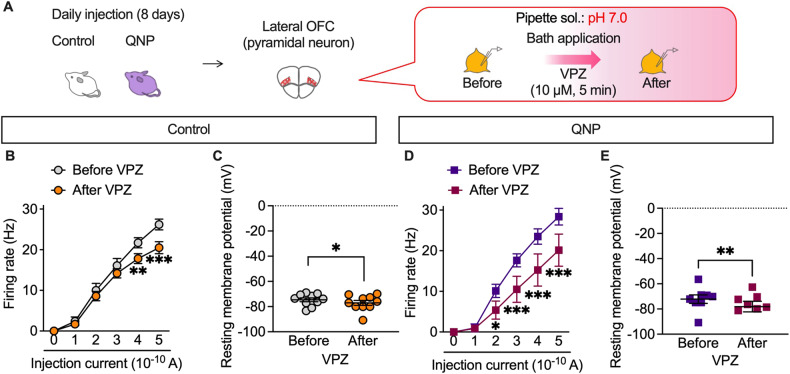
Vonoprazan attenuated neuronal activity in the lateral OFC under acidic intracellular conditions. **A**–**E** The firing responses induced by current injection (0–500 pA; 1 s duration) were recorded before and after bath application of vonoprazan (VPZ; 10 μM) in intracellular pH 7.0 in the lateral OFC pyramidal neurons obtained from control (**B**, **C**) and QNP-treated mice (**D**, **E**). **B**, **D** Firing responses induced by current injection; **C**, **E** the resting membrane potential. *P < 0.05; **P < 0.01; ***P < 0.001.

P-type proton pumps in cortical neurons are involved in the homoeostatic maintenance of intracellular pH and membrane excitability modulation. We recorded firing activity under slightly acidic intracellular conditions (pH 7.0) to confirm whether the inhibitory effects of vonoprazan on neuronal activity were facilitated by weak intracellular acidosis (Fig. 6A). At pH 7.0, vonoprazan significantly decreased the firing activity of lateral OFC pyramidal neurons in control mice (Fig. 6B, C). Vonoprazan significantly decreased the firing activity in the late phase of the depolarising pulse (Supplementary Fig. 2). These inhibitory effects of vonoprazan were similarly observed in QNP-treated mice. Additionally, there was significant inhibition at lower injection currents (200 and 300 pA) than in normal pH conditions (Fig. 6D, E). In control and QNP-treated mice, the combination of intracellular acidic conditions and vonoprazan treatment caused slight but significant hyperpolarisation of the resting membrane potential. These results further indicated that vonoprazan inhibited the firing activity of lateral OFC pyramidal neurons through intracellular proton accumulation.

The preclinical experiment results supported the data-driven hypothesis that PPIs inhibited OCD-like symptoms.

## Discussion

In our study, two clinical big datasets analyses revealed that PPIs mitigate the risk of OCD-like symptoms induced by D_2_R agonists. Moreover, vonoprazan administration inhibited OCD-like behaviours and ameliorated lateral OFC hyperactivity in QNP-treated mice through intracellular acidification.

Clinical reports have shown a relationship between the use of dopaminergic agents and impulsive/compulsive symptoms [41, 42,43, 44]. We have confirmed this hypothesis through chronological analysis. OCD-like symptoms induced by D_2_R agonists have been reported in patients with Parkinson’s disease, restless legs syndrome, and hyperprolactinemia. D_2_R abnormalities occur in psychiatric patients with obsession and compulsion (e.g. OCD [1], eating disorder [44], and substance abuse [45]). Our findings demonstrated that repeated stimulation of D_2_R causes OCD-like symptoms regardless of the primary disease.

FAERS and MarketScan data analyses revealed that PPIs could be therapeutic agents for OCD-like symptoms with or without the use of D_2_R agonists. PPIs can penetrate the blood–brain barrier [46]. Since the human brain expresses the *Atp4a* gene [47], PPIs can affect the central nervous system despite typically acting in the peripheral organs.

In QNP-treated mice, there was a difference in the treatment duration and onset of inhibitory effects on repeated chewing behaviour between lansoprazole and vonoprazan. Since lansoprazole requires acid-induced structural change for activation, this process may become a rate-limiting step in the brain, which has a higher pH than the stomach [48]. Compared with conventional PPIs (e.g. lansoprazole), vonoprazan has a unique molecular framework and differences in the action mechanisms [27], including reversible inhibition of P-type proton pumps through prevention of K^+^ binding, no requirement of the activation process, and high stability under acidic conditions. These differences may contribute to the acute effects of vonoprazan on repetitive behaviour.

Hyperactivity in the OFC is related to OCD-like symptoms. In patients with OCD, there is a positive correlation between neural activity in the lateral OFC and symptom severity [35]. Moreover, chronic administration of SSRIs normalises the lateral OFC activity only in SSRI-responsive patients with OCD [36]. Consistent with these reports, we previously found that QNP-treated mice exhibit hyperactivity in the lateral OFC pyramidal neurons, which was suppressed by the 4-week administration of SSRI [12, 17]. In this study, a single administration of vonoprazan successfully decreased the neural activity of lateral OFC pyramidal neurons. These acute inhibitory effects of vonoprazan on neural activity in the lateral OFC may result in its rapid therapeutic efficacy against OCD-like behaviours.

In patients with OCD, symptoms can be classified into several subtypes known as symptom dimensions, which show different therapeutic responses toward medication [49, 50]. Consistent with these reports, 4-week SSRI administration in QNP-treated mice improved maladaptive habits. However, it did not affect repetitive behaviour [12], suggesting that it is an ‘SSRI-resistant’ OCD-like behaviour. Since vonoprazan inhibited both repetitive and maladaptive behaviours, our findings indicate that vonoprazan is a rapid-acting, effective treatment for SSRI-resistant symptoms.

Since both vonoprazan and long-term SSRI administration reduced the firing activity of the lateral OFC pyramidal neurons, differences in their behaviour modulation require clarification. The differences could be attributed to differences in the mechanisms of action in the lateral OFC. We previously demonstrated that in the lateral OFC pyramidal neurons of QNP-treated mice, there is an increase and decrease in excitatory and inhibitory inputs, respectively [12, 17]. Chronic SSRI use improves this abnormal excitatory/inhibitory balance by potentiating the inhibitory tone [17].

In contrast, vonoprazan directly acts on pyramidal neurons and inhibits depolarisation-induced action potential generation, showing a more potent inhibitory effect even under potentiated excitatory inputs. Additionally, this data did not exclude the effects of vonoprazan on excitatory inputs from outside the lateral OFC or interneurons within the lateral OFC. Since abnormalities in both excitatory and inhibitory inputs in the lateral OFC have been reported in mice with OCD-related dysfunctions [12, 17, 51], future studies investigating the effects on excitatory/inhibitory balance are necessary to elucidate the therapeutic potential of vonoprazan and conventional PPIs.

Although we observed that vonoprazan and *Atp4a* KD only slightly decreased the intracellular pH, such manipulations successfully reduced neural excitability. One possible reason for such a small effect may be the recording condition. A tetrodotoxin-induced suppression of firing activity was used for pH measurements to assess the effects of vonoprazan and *Atp4a* KD on the intracellular pH independent of their effects on firing activity. However, as neural activity, such as excitatory inputs via NMDA receptors and depolarisation, can transiently lower intracellular pH [21, 52, 53], tetrodotoxin might decrease proton influx and reduce the effects of blockade of proton efflux via proton pumps. Additionally, neural activity-induced transient acidification is more prominent in dendrites than in the soma region. The previous report demonstrates that the current injection-induced activity of Purkinje cells can decrease dendritic pH up to 0.3 units. In comparison, average acidification in the soma regions is around 0.03 pH units [54]. This finding also supports our hypothesis that, in the presence of neural activity, transient pH drop can be larger, at least locally, than observed here. However, further studies are needed to elucidate the actual pH shift induced in the presence of neuronal activity.

The exact mechanisms underlying acidification-induced activity modulation in neurons remain unclear, whereas numerous molecules alter their function in response to intracellular H^+^ in the brain [55]. Some channels may be partly involved in modulating neural activity through both toxic acidosis and physiological pH reduction, including tandem pore domain weak inward rectifier K^+^ channel-related K^+^ channels (TREKs) [56–58] and high-voltage-activated voltage-dependent Ca^2+^ channels [59–63]. These observations suggest that a slight decrease in intracellular pH modulates pyramidal neuron activity in the lateral OFC. However, further studies are required to elucidate the detailed mechanisms through which vonoprazan inhibits neuronal activity through intracellular acidification.

In conclusion, this study demonstrated the efficacy of PPIs against OCD-like symptoms using data mining prediction with experimental validation. Among the PPIs, vonoprazan showed rapid and effective action against experimentally induced OCD-like behaviours. In the lateral OFC, vonoprazan inhibited neuronal hyperactivity, which could have been through intracellular acidification. These results provide new insights into the therapeutic strategies for OCD.

### Supplementary information

Supplementary data

Supplementary tables 2 & 3

Supplementary methods

Statistical information

## Data Availability

FAERS adverse event reports were obtained from the FDA website (http://www.fda.gov/Drugs/GuidanceComplianceRegulatoryInformation/Surveillance/AdverseDrugEffects/). MarketScan’s data use agreements do not permit the sharing of source data or data derivatives with individuals and institutions not covered by the agreement. These data sources may be accessed by other researchers through their own data use agreements (https://www.ibm.com/products/marketscan-research-databases/databases).

## Code availability

We used open-source program languages mysql (version 8.0.23) and R (version 4.2.1) to analyse data. Source codes are available upon request.
