# Supplementary material for: Amelioration of obsessive-compulsive disorder by intracellular acidification of cortical neurons with a proton pump inhibitor
Source: Transl Psychiatry. 2024 Jan 16;14:27. doi: 10.1038/s41398-024-02731-3 (PMC10791614; doi:10.1038/s41398-024-02731-3)
Supplement: Supplementary file 1 — Supplementary data [file 41398_2024_2731_MOESM1_ESM.docx]

**
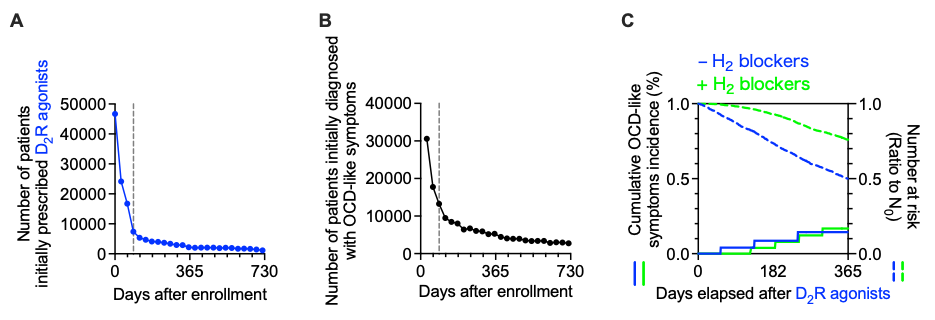
Supplementary Fig. 1: Time distribution of the first event after enrolment and the effect of concomitant use of H_2_ blockers on the incidence of OCD-like symptoms induced by D_2_R agonists in the MarketScan data.**

Time intervals from insurance enrolment to the first prescription of D_2_R agonists (pramipexole or ropinirole) (**A**) and the initial diagnosis of OCD-like symptoms (**B**) were analysed. The number of patients was shown daily. From these data, patients enrolled in the health insurance for 90 days were excluded from the subsequent analysis to allow a run-in period. (**C**) Occurrence of OCD-like symptoms within one year of D_2_R agonists administration. In MarketScan data, Kaplan–Meier curves for the cumulative incidence ratio of OCD-like symptoms in D_2_R agonists users are shown for each population: one without (red) and one with (blue) co-prescription of H_2_ blockers. The dotted lines show the number of patients at risk as a ratio to the initial number (N_0_) of patients.

**
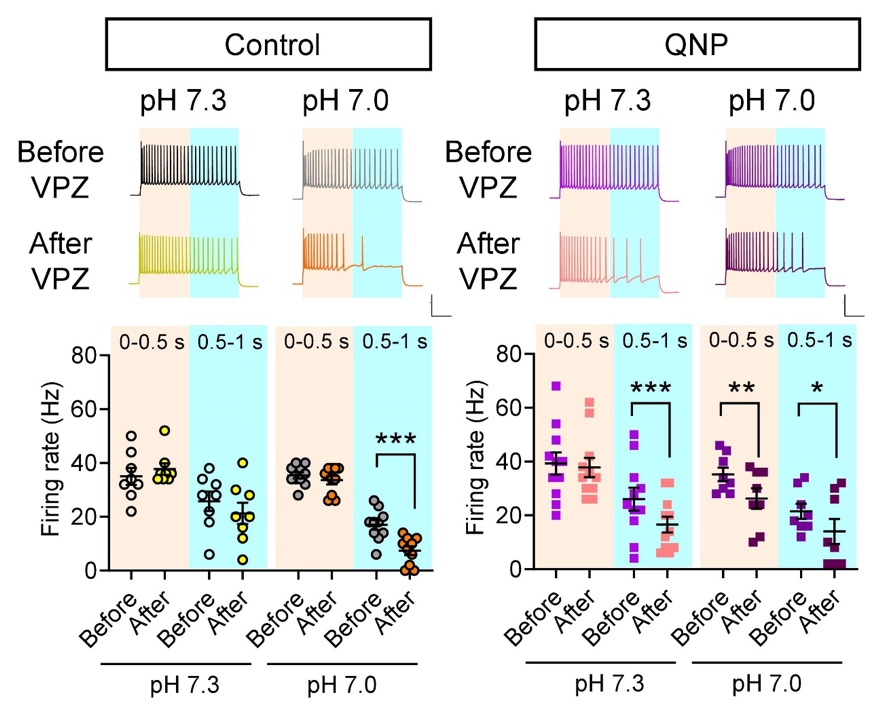
Supplementary Fig. 2: Distinct effects of VPZ on firing activity in the early and late phase of depolarization.**

Firing rates of lOFC pyramidal neurons in the early (0–0.5 s) and late (0.5–1 s) phase of 1-s depolarizing pulse (500 pA; firing rates in 1-s pulse are shown in Fig 5E, 5G, 7B & 7D). (**Left**) Firing activity of pyramidal neurons from control mice before/after application of VPZ (10 μM). pH 7.3: Two-way repeated measures ANOVA; VPZ *F*(1, 7) = 0.119, *P* = 0.740; Phase *F*(1, 7) = 25.44, *P* = 0.001; Interaction *F*(1, 7) = 9.45, *P* = 0.018. pH 7.0: Two-way repeated measures ANOVA; VPZ *F*(1, 9) = 10.91, *P* = 0.009; Phase *F*(1, 9) = 286.7, *P* < 0.0001; Interaction *F*(1,9) = 30.49, *P* = 0.0004. (**Right**) Firing activity of pyramidal neurons from QNP-treated mice before/after application of VPZ (10 μM). pH 7.3: Two-way repeated measures ANOVA; VPZ *F*(1, 10) = 10.60, *P* = 0.009; Phase *F*(1, 10) = 159.2, *P* < 0.0001; Interaction *F*(1,10) = 17.60, *P* = 0.002. pH 7.0: Two-way repeated measures ANOVA; VPZ *F*(1, 7) = 6.555, *P* = 0.037; Phase *F*(1, 7) = 20.48, *P* = 0.0027; Interaction *F*(1, 7) = 0.2432, *P* = 0.637. Scale bar = 200 ms and 50 mV

**Supplementary Table 1: OCD-like symptoms defined using preferred terms (PT) in FAERS data.**

| **PT** | **Number of patients** |
| --- | --- |
| Obsessive-compulsive disorder | 5,674 |
| Compulsive shopping | 1,547 |
| Obsessive thoughts | 1,281 |
| Compulsive sexual behaviour | 650 |
| Trichotillomania | 428 |
| Compulsions | 419 |
| Compulsive hoarding | 281 |
| Obsessive-compulsive symptom | 122 |
| Compulsive lip biting | 60 |
| Obsessive rumination | 37 |
| Compulsive cheek biting | 20 |
| Compulsive handwashing | 18 |

**Supplementary Table 2:** **Overall results of disproportionality analysis for OCD-like symptoms in FAERS dataset.**

**Supplementary Table 3: Overall confounding effects of concomitant drugs on D_2_R agonists-induced OCD-like symptoms in FAERS dataset.**

(Supplementary Tables 2 and 3 are submitted in a separate Excel file.)

**Supplementary Table 4: D_2_R agonists defined in MarketScan data.**

| **Drug** | **Dosage form** | **Number of patients** |
| --- | --- | --- |
| Pramipexole Dihydrochloride | Oral | 65,937 |
| Ropinirole Hydrochloride | Oral | 101,833 |

**Supplementary Table 5: Population characteristics of proton pump inhibitors in MarketScan data.**

| **Drug** | **Dosage form** | **Number of patients** |
| --- | --- | --- |
| Omeprazole | Oral | 1,866,889 |
| Pantoprazole Sodium | Oral | 1,127,643 |
| Esomeprazole Magnesium | Oral | 280,157 |
| Lansoprazole | Oral | 154,310 |
| Dexlansoprazole | Oral | 88,083 |
| Rabeprazole Sodium | Oral | 39,442 |
| Esomeprazole Magnesium/Naproxen | Oral | 21,619 |
| Amoxicillin;Clarithromycin;Lansoprazole | Oral | 15,596 |
| Omeprazole/Sodium Bicarbonate | Oral | 9,958 |
| Omeprazole Magnesium | Oral | 1,502 |
| Amoxicillin;Clarithromycin;Omeprazole | Oral | 1,285 |
| Omeprazole | Route Not Applicable | 492 |
| Esomeprazole Strontium | Oral | 220 |
| Aspirin/Omeprazole | Oral | 160 |
| Lansoprazole | Route Not Applicable | 90 |
| Pantoprazole Sodium | Intravenous | 74 |
| Esomeprazole Sodium | Intravenous | 6 |
| Omeprazole;Powder, Multi Ingredient | Route Not Applicable | 3 |
| Pantoprazole Sodium | Route Not Applicable | 2 |

**Supplementary Table 6: OCD-like symptoms defined by ICD-10 in MarketScan data.**

| **Symptom** | **ICD-10** | **Number of patients** |
| --- | --- | --- |
| Obsessive-compulsive disorder | F42 | 36,092 |
| Mixed obsessional thoughts and acts | F422 | 51,797 |
| Hoarding disorder | F423 | 1,706 |
| Excoriation (skin-picking) disorder | F424 | 8,375 |
| Other obsessive-compulsive disorder | F428 | 15,619 |
| Obsessive-compulsive disorder, unspecified | F429 | 80,201 |
| (Non-Billable Dx) Habit and impulse disorders | F63 | 12 |
| Trichotillomania | F633 | 10,579 |
| (Non-Billable Dx) Other habit and impulse disorders | F638 | 14 |
| Other habit and impulse disorders | F6389 | 2,527 |
| Habit and impulse disorder, unspecified | F639 | 10,145 |

**Supplementary Table 7: OCD-like symptoms defined by ICD-10 in MarketScan data.**

| **Confounding factors** | **Definition** |
| --- | --- |
| Age | ≥ 65 years |
| Sex | – |
| SSRIs | citalopram, escitalopram, fluoxetine, fluvoxamine, paroxetine, sertraline |
| H_2_ blockers | cimetidine, famotidine, nizatidine, ranitidine |
| Parkinson’s disease | ICD-10 code: G20 |
| Restless legs | ICD-10 code: G2581 |
| Diseases of the oesophagus, stomach, and duodenum | ICD-10 code: K20–29 |
| Diseases of the liver | ICD-10 code: K7 |
| Renal failure | ICD-10 code: N17–19 |
| Hyperprolactinemia | ICD-10 code: E22 |

**Supplementary Table 8:** **Population characteristics of patients taking D_2_R agonists with or without proton pump inhibitors before propensity score matching in MarketScan data.**

|  | **Without PPIs** | | **With PPIs** | | ***P value*** |
| --- | --- | --- | --- | --- | --- |
| Number of patients | 46,278 | 100% | 5,531 | 100% |  |
| OCD-like symptoms (day <= 365) | 158 | 0.341% | 5 | 0.090% |  |
| Median age (IQR) | 53  (44–61) |  | 54  (45–61) |  |  |
| Elderly (over 65) | 6,368 | 13.8% | 909 | 16.4% | 7.07*10^-8^ |
| Female | 28,044 | 60.6% | 3,758 | 67.9% | < 2.20*10^-16^ |
| Concomitant drug |  |  |  |  |  |
| SSRI | 16,585 | 35.8% | 2,323 | 42.0% | < 2.20*10^-16^ |
| H_2_ blocker | 2,870 | 6.20% | 973 | 17.6% | < 2.20*10^-16^ |
| Comorbidity |  |  |  |  |  |
| Parkinson disease | 2,824 | 6.10% | 251 | 4.54% | 3.78*10^-6^ |
| Restless legs | 26,343 | 56.9% | 3,416 | 61.8% | 6.75*10^-12^ |
| Diseases of oesophagus, stomach and duodenum | 7,833 | 16.9% | 3,688 | 66.7% | < 2.20*10^-16^ |
| Diseases of liver | 3,150 | 6.81% | 901 | 16.3% | < 2.20*10^-16^ |
| Renal failure | 3,395 | 7.34% | 672 | 12.1% | < 2.20*10^-16^ |

**Supplementary Table 9: Population characteristics of patients taking D_2_R agonists with or without OCD-like symptoms before propensity score matching to analyse the concomitant use effects of proton pump inhibitors in MarketScan data.**

|  | **Without OCD-like symptoms** | | **With OCD-like symptoms** | | ***P value*** |
| --- | --- | --- | --- | --- | --- |
| Number of patients (day <= 365) | 51,646 | 100% | 163 | 100% |  |
| Elderly (over 65) | 7,263 | 14.1% | 14 | 16.4% | 0.058 |
| Female | 31,708 | 61.4% | 94 | 67.9% | 0.371 |
| Concomitant drug |  |  |  |  |  |
| SSRI | 18,817 | 36.4% | 91 | 42.0% | 4.34*10^-7^ |
| H_2_ blocker | 6,080 | 11.8% | 14 | 17.6% | 0.255 |
| Comorbidity |  |  |  |  |  |
| Parkinson disease | 3,065 | 5.93% | 10 | 4.54% | 1.00 |
| Restless legs | 29,686 | 57.5% | 73 | 61.8% | 0.001 |
| Hyperprolactinemia | 142 | 0.275% | 4 | 2.45% | 1.61*10^-7^ |
| Diseases of oesophagus, stomach and duodenum | 11,500 | 22.3% | 21 | 66.7% | 0.005 |
| Diseases of liver | 4,039 | 7.82% | 12 | 16.3% | 0.943 |
| Renal failure | 4,058 | 7.86% | 9 | 12.1% | 0.336 |

**Supplementary Table 10: Population characteristics of patients taking D_2_R agonists with or without proton pump inhibitors after propensity score matching in MarketScan data.**

|  | **Without PPIs** | | **With PPIs** | | ***P value*** |
| --- | --- | --- | --- | --- | --- |
| Number of patients | 5,528 | 100% | 5,528 | 100% |  |
| OCD-like symptoms (day <= 365) | 25 | 0.452% | 5 | 0.090% |  |
| Median age (IQR) | 55  (46–62) |  | 54  (45–61) |  |  |
| Elderly (over 65) | 932 | 16.9% | 909 | 16.4% | 0.574 |
| Female | 3,761 | 66.0% | 3,755 | 67.9% | 0.640 |
| Concomitant drug |  |  |  |  |  |
| SSRI | 2,353 | 42.6% | 2,320 | 42.0% | 0.538 |
| H_2_ blocker | 915 | 16.6% | 865 | 15.6% | 0.205 |
| Comorbidity |  |  |  |  |  |
| Parkinson disease | 228 | 4.12% | 251 | 4.54% | 0.304 |
| Restless legs | 3,436 | 62.2% | 3,413 | 61.7% | 0.667 |
| Diseases of oesophagus, stomach and duodenum | 3,685 | 66.7% | 3,685 | 66.7% | 1.00 |
| Diseases of liver | 855 | 15.5% | 898 | 16.2% | 0.274 |
| Renal failure | 688 | 12.4% | 669 | 12.1% | 0.602 |

**Supplementary Table 11: Population characteristics of patients taking D_2_R agonists with or without OCD-like symptoms after propensity score matching of the cohorts in analysing the concomitant use effects of proton pump inhibitors in MarketScan data.**

|  | **Without OCD-like symptoms** | | **With OCD-like symptoms** | | ***P value*** |
| --- | --- | --- | --- | --- | --- |
| Number of patients  (day <= 365) | 11,035 | 100% | 21 | 100% |  |
| Elderly (over 65) | 1,840 | 16.7% | 1 | 4.76% | 0.236 |
| Female | 7,471 | 67.7% | 15 | 71.4% | 0.896 |
| Concomitant drug |  |  |  |  |  |
| SSRI | 4,657 | 42.2% | 16 | 76.2% | 3.40*10^-3^ |
| H_2_ blocker | 1,777 | 16.1% | 3 | 14.3% | 1.00 |
| Comorbidity |  |  |  |  |  |
| Parkinson disease | 479 | 4.34% | 0 | 0% | 1.00 |
| Restless legs | 6,839 | 62.0% | 10 | 47.6% | 0.259 |
| Hyperprolactinemia | 43 | 0.390% | 1 | 4.76% | 0.080 |
| Diseases of oesophagus, stomach and duodenum | 7,358 | 66.7% | 12 | 57.1% | 0.487 |
| Diseases of liver | 1,750 | 15.9% | 3 | 14.3% | 1.00 |
| Renal failure | 1,354 | 12.3% | 3 | 14.3% | 1.00 |

**Supplementary Table 12: Population characteristics of H_2_ blockers in MarketScan data.**

| **Drug** | **Dosage form** | **Number of patients** |
| --- | --- | --- |
| Ranitidine Hydrochloride | Oral | 692,834 |
| Famotidine | Oral | 378,331 |
| Famotidine/Ibuprofen | Oral | 46,032 |
| Cimetidine | Oral | 16,555 |
| Nizatidine | Oral | 6,297 |
| Cimetidine Hydrochloride | Oral | 4,898 |
| Famotidine | Intravenous | 158 |
| Ranitidine Hydrochloride | Injection | 42 |
| Cimetidine | Route Not Applicable | 37 |
| Ranitidine Hydrochloride | Route Not Applicable | 20 |
| Famotidine | Route Not Applicable | 5 |
| Calcium Carbonate/Famotidine/Magnesium Hydroxide | Oral | 2 |

**Supplementary Table 13: Population characteristics of patients taking D_2_R agonists with or without H_2_ blockers before propensity score matching in MarketScan data.**

|  | **Without H_2_ blockers** | | **With H_2_ blockers** | | ***P value*** |
| --- | --- | --- | --- | --- | --- |
| Number of patients | 64,787 | 100% | 2,682 | 100% |  |
| OCD-like symptoms (day <= 365) | 203 | 0.313% | 4 | 0.149% |  |
| Median age (IQR) | 54  (45–61) |  | 55  (46–62) |  |  |
| Elderly (over 65) | 9,875 | 15.2% | 517 | 19.3% | 1.66*10^-8^ |
| Female | 40,095 | 61.9% | 891 | 70.5% | < 2.20*10^-16^ |
| Concomitant drug |  |  |  |  |  |
| SSRI | 24,355 | 37.6% | 1,195 | 44.6% | 3.72*10^-13^ |
| Proton pump inhibitor | 21,376 | 33.0% | 1,694 | 63.2% | < 2.20*10^-16^ |
| Comorbidity |  |  |  |  |  |
| Parkinson disease | 3,591 | 6.10% | 109 | 4.06% | 1.14*10^-3^ |
| Restless legs | 37,252 | 57.5% | 1,658 | 61.8% | 9.98*10^-6^ |
| Hyperprolactinemia | 169 | 0.261% | 18 | 0.671% | 1.61*10^-4^ |
| Diseases of oesophagus, stomach and duodenum | 20,702 | 32.0% | 1,871 | 69.8% | < 2.20*10^-16^ |
| Diseases of liver | 5,959 | 9.20% | 458 | 17.1% | < 2.20*10^-16^ |
| Renal failure | 5,674 | 8.76% | 361 | 13.5% | < 2.20*10^-16^ |

**Supplementary Table 14: Population characteristics of patients taking D_2_R agonists with or without OCD-like symptoms before propensity score matching to analyse the concomitant use effects of H_2_ blockers in MarketScan data.**

|  | **Without OCD-like symptoms** | | **With OCD-like symptoms** | | ***P value*** |
| --- | --- | --- | --- | --- | --- |
| Number of patients (day <= 365) | 67,261 | 100% | 207 | 100% |  |
| Elderly (over 65) | 10,375 | 15.4% | 17 | 8.21% | 0.006 |
| Female | 41,862 | 62.2% | 124 | 59.9% | 0.535 |
| Concomitant drug |  |  |  |  |  |
| SSRI | 25,424 | 37.8% | 126 | 60.9% | 1.37*10^-11^ |
| Proton pump inhibitor | 23,013 | 34.2% | 57 | 27.5% | 5.13*10^-2^ |
| Comorbidity |  |  |  |  |  |
| Parkinson disease | 3,686 | 5.5% | 14 | 6.76% | 0.511 |
| Restless legs | 38,816 | 57.7% | 94 | 45.4% | 4.56*10^-4^ |
| Hyperprolactinemia | 182 | 0.271% | 5 | 2.42% | 2.98*10^-4^ |
| Diseases of oesophagus, stomach and duodenum | 22,526 | 33.5% | 47 | 22.7% | 1.33*10^-3^ |
| Diseases of liver | 6,398 | 9.51% | 19 | 9.18% | 0.964 |
| Renal failure | 6,022 | 8.95% | 13 | 6.28% | 0.221 |

**Supplementary Table 15: Population characteristics of patients taking D_2_R agonists with or without H_2_ blockers after propensity score matching in MarketScan data.**

|  | **Without H_2_ blockers** | | **With H_2_ blockers** | | ***P value*** |
| --- | --- | --- | --- | --- | --- |
| Number of patients | 2,680 | 100% | 2,680 | 100% |  |
| OCD-like symptoms (day <= 365) | 3 | 0.112% | 4 | 0.149% |  |
| Median age (IQR) | 55 (47–62) |  | 55 (46–62) |  |  |
| Elderly (over 65) | 517 | 19.3% | 515 | 19.2% | 0.972 |
| Female | 1,911 | 71.3% | 1,890 | 70.5% | 0.548 |
| Concomitant drug |  |  |  |  |  |
| SSRI | 1,225 | 45.7% | 1,194 | 44.6% | 0.410 |
| Proton pump inhibitor | 1,663 | 62.1% | 1,692 | 63.1% | 0.429 |
| Comorbidity |  |  |  |  |  |
| Parkinson disease | 112 | 4.18% | 108 | 4.03% | 0.836 |
| Restless legs | 1,664 | 62.1% | 1,656 | 61.8% | 0.844 |
| Hyperprolactinemia | 17 | 0.634% | 17 | 0.634% | 1.00 |
| Diseases of oesophagus, stomach and duodenum | 1,874 | 69.9% | 1,869 | 69.8% | 0.905 |
| Diseases of liver | 456 | 17.0% | 457 | 17.1% | 1.00 |
| Renal failure | 348 | 13.0% | 360 | 13.4% | 0.657 |

**Supplementary Table 16: Population characteristics of patients taking D_2_R agonists with or without OCD-like symptoms after propensity score matching of the cohorts in analysing the concomitant use effects of H_2_ blockers in MarketScan data.**

|  | **Without OCD-like symptoms** | | **With OCD-like symptoms** | | ***P value*** |
| --- | --- | --- | --- | --- | --- |
| Number of patients (day <= 365) | 5,353 | 100% | 7 | 100% |  |
| Elderly (over 65) | 1,031 | 19.3% | 1 | 14.3% | 1.00 |
| Female | 3,796 | 70.9% | 5 | 71.4% | 1.00 |
| Concomitant drug |  |  |  |  |  |
| SSRI | 2,416 | 45.1% | 3 | 42.9% | 1.00 |
| Proton pump inhibitor | 3,353 | 62.6% | 2 | 28.6% | 0.111 |
| Comorbidity |  |  |  |  |  |
| Parkinson disease | 220 | 4.11% | 0 | 0% | 1.00 |
| Restless legs | 3,315 | 61.9% | 5 | 71.4% | 0.898 |
| Hyperprolactinemia | 33 | 0.616% | 1 | 14.3% | 0.044 |
| Diseases of oesophagus, stomach and duodenum | 3,739 | 69.8% | 4 | 57.1% | 0.438 |
| Diseases of liver | 911 | 17.0% | 2 | 28.6% | 0.341 |
| Renal failure | 707 | 13.2% | 1 | 14.3% | 1.00 |

**Supplementary Table 17: Daily and cumulative doses and administration periods of D_2_R agonists, levodopa, and proton pump inhibitors in propensity score-matched cohorts when used in combination with H_2_ blockers of MarketScan data using L-dopa equivalent dose (LED) and omeprazole equivalent dose (OED).**

|  |  | **Without H_2_ blockers**  (n = 2,680) | **Without H_2_ blockers**  (n = 2,680) | ***P value*** | ***T value*** |
| --- | --- | --- | --- | --- | --- |
| D_2_R agonists | Average daily dose (LED, median&IQR) | 20.0  (10.0–49.2) | 21.9  (10.0–50.0) | 0.164 | t(3644) = 1.39 |
|  | Cumulative dose (LED, median&IQR) | 2,700  (750–9,000) | 3,750  (1,050–13,500) | 0.097 | t(3599) = 1.66 |
|  | Administration period (day, median&IQR) | 120 (30–300) | 180 (60–450) | < 0.001 | t(5186) = 8.75 |
| Levodopa | Average daily dose (LED, median&IQR) | 305 (269–526) | 382 (200–799) | 0.777 | t(196) = 0.284 |
|  | Cumulative dose (LED, median&IQR) | 106,830  (27,000–270,750) | 210,000  (36,000–565,450) | 0.913 | t(206) = 0.109 |
|  | Administration period (day, median&IQR) | 270 (101–608) | 450 (128–887) | 0.189 | t(215) = 2.37 |
| PPIs | Average daily dose (OED, median&IQR) | 26.6  (20.0–40.0) | 26.6  (20.0–40.0) | 0.774 | t(3331) = 0.338 |
|  | Cumulative dose (OED, median&IQR) | 9,600  (3,600–20,400) | 9,900  (3,600–21,600) | 0.482 | t(1955) = 0.703 |
|  | Administration period (day, median&IQR) | 360 (90–720) | 330 (120–720) | 0.735 | t(2764) = 0.287 |

**Supplementary Table 18:** **Population characteristics of non-D_2_R agonists users with or without proton pump inhibitors before propensity score matching in MarketScan data.**

|  | **Without PPIs** | | **With PPIs** | | ***P value*** |
| --- | --- | --- | --- | --- | --- |
| Number of patients | 37,506,560 | 100% | 3,151,835 | 100% |  |
| OCD-like symptoms (day <= 365) | 104,312 | 0.278% | 5,744 | 0.182% |  |
| Median age (IQR) | 35  (18–51) |  | 51  (38–59) |  |  |
| Elderly (over 65) | 1,111,710 | 2.96% | 399,011 | 12.7% | < 2.20*10^-16^ |
| Female | 14,172,215 | 37.8% | 1,801,957 | 57.2% | < 2.20*10^-16^ |
| Concomitant drug |  |  |  |  |  |
| SSRI | 2,146,321 | 5.72% | 565,826 | 18.0% | < 2.20*10^-16^ |

**Supplementary Table 19: Population characteristics of non-D_2_R agonists users with or without proton pump inhibitors after propensity score matching in MarketScan data.**

|  | **Without PPIs** | | **With PPIs** | | ***P value*** |
| --- | --- | --- | --- | --- | --- |
| Number of patients | 154,939 | 100% | 154,939 | 100% |  |
| OCD-like symptoms (day <= 365) | 654 | 0.422% | 289 | 0.187% |  |
| Median age (IQR) | 40  (23–57) |  | 51  (39–60) |  |  |
| Elderly (over 65) | 19,561 | 12.6% | 19,561 | 12.6% | 1.00 |
| Female | 88,801 | 57.3% | 88,801 | 57.3% | 1.00 |
| Concomitant drug |  |  |  |  |  |
| SSRI | 36,144 | 23.3% | 36,144 | 23.3% | 1.00 |
