# Supplementary material for: Amelioration of obsessive-compulsive disorder by intracellular acidification of cortical neurons with a proton pump inhibitor
Source: Transl Psychiatry. 2024 Jan 16;14:27. doi: 10.1038/s41398-024-02731-3 (PMC10791614; doi:10.1038/s41398-024-02731-3)
Supplement: Supplementary file 3 — Supplementary methods [file 41398_2024_2731_MOESM3_ESM.docx]

**Supplementary Methods**

*The definition of run-in period*

To set the time distribution of the first event after enrolment in MarketScan data, we defined the run-in period to detect the onset and causality of OCD-like symptoms induced by D_2_R agonists by eliminating patients with a diagnosis of OCD-like symptoms or a prescription with D_2_R agonists before insurance enrolment. Accordingly, the number of patients with an initial prescription of D_2_R agonists and diagnosis of OCD-like symptoms (Supplementary Fig. 1A–B) was much higher during the first three months, and stabilised after four months. After excluding the aforementioned patients during the first three months (run-in period), patients who received D_2_R agonists were included in the cohort analysis.

*Reagents*

QNP (dopamine D_2_ agonist; Tocris Bioscience, Bristol, UK) was dissolved in saline. Lansoprazole (PPI; Tokyo Chemical Industry, Tokyo, Japan), vonoprazan (active potassium-competitive acid blocker; Struchem, Wujiang, China), and nigericin (high-affinity ionophore for monovalent cations such as H^+^; Cayman, Ann Arbor, MI, USA) were dissolved in dimethyl sulfoxide (DMSO). Tetrodotoxin (a voltage-dependent Na^+^ channel blocker; Sigma-Aldrich, St. Louis, MO) and citalopram (SSRI; Struchem) were dissolved in water. Stock solutions were stored at −20°C until use, followed by dissolving in saline, 20 % Tween80 solution (for lansoprazole), artificial cerebrospinal fluid (ACSF), or Krebs–Ringer solution for use in experiments. The final concentration of DMSO was < 5% and < 0.05% for i.p./i.c.v. injection and electrophysiology experiments, respectively.

*Stereotaxic surgery and microinjection*

As reported previously [13], a guide cannula was inserted into the right lateral ventricle at the OFC (AP=−0.3 mm, ML=+1.0 mm, DV=+3.5 mm from the bregma). The experimental procedure was started at ≥ 7 postoperative days, and vonoprazan (3 μg) was injected at 0.6 μL/min; the injection cannula was left in place for 5 min.

*Confirmation of brain regions implanted guide cannula*

After the experiments, 3 μL of Evans Blue solution was injected through the cannula to confirm the injection site. Animals whose injection site was incorrect were excluded from the analyses.

*Open-field test*

Each mouse was placed at the centre of an open-field apparatus (75 × 75 cm). Locomotor activity was monitored for 10 min immediately after i.c.v. injection of vonoprazan. The total distance travelled was recorded and analysed using a video tracking system (ANY-maze version 4.99).

*Real-time qRT-PCR*

Real-time qPCR was performed as previously described [13]. The oligonucleotide primers used were as follows: 5′-CTTACTAATTGGAGGCCATGTAG -3′ and 5′- TGTCAAGCTCATTTCCTGGT -3′ for *Gapdh*, 5′-GCTGGTTCCCTCTGTTGTGT -3′ and 5′- TACAGGCGCTGACCAAATGT-3′ for *Atp4a*.

*Immunostaining*

Immunostaining was performed as previously described [17]. We used rabbit polyclonal anti-c-Fos antibody (1:2000; 9F6; Cell Signalling Technology, Beverly, MA) as primary antibody; Alexa Fluor 594-labelled donkey anti-rabbit IgG as the secondary antibody (1:200; Life Technologies, Carlsbad, CA). Labelled sections were mounted on glass slides using DAPI Fluoromount-G. The number of c-Fos-positive cells in the lateral OFC (AP +2.7 mm from the bregma) was counted in six sections (0.045 mm^2^/section) per mouse.

*miRNA targeting sequence*

The miRNA targeting sequence was 5′- GAGACCGTTTAAACGGTCTCA -3′ for negative control (NC) and 5′- AAAGATATCTGTGCAGAGTTCGTTTTGGCCACTGACTGACGAACTCTGCAGATATCTTT -3′ for *Atp4a*.

*Preparation of AAV vector*

As previously described [7], Lenti-X 293T cells (632180; Clontech) were transfected using pAAV-hSyn-mCherry-SEpHluorin, pAAV-DJ (VPK-402-DJ; Cell Biolabs), and pHelper (240202; Cell Biolabs) using polyethyleneimine (‘Max’, Polysciences, Warrington, PA, USA). After 60–72 h transfection, the cells were gently collected and freeze-thawed four times to break the cell membrane. DNA and RNA were removed using benzonase nuclease (Sigma-Aldrich). The lysate was incubated at 45°C for 15 minutes, centrifuged at 17,765 × *g* for 10 minutes, and the supernatant collected.

*Primary cortex neuronal culture*

Primary cultures were prepared using cortical neurons obtained from C57BL/6J mice (E14–E16), as previously described with some modifications [13]. Cells were plated on coverslips or culture plates coated with poly-d-lysine (Sigma Millipore) and maintained in Neurobasal Plus medium (Thermo Fisher Scientific) containing 2% B-27 Plus supplement (Thermo Fisher Scientific) and 1% penicillin/streptomycin/glutamine mixed solution (Nacalai Tesque) placed in an incubator with 5% CO_2_ at 37°C. Half of the culture medium was replaced with fresh medium every 2–3 days.

After cultivation for two days, the cells to be used for measurement of intracellular pH and *Atp4a* knockdown experiments were treated with AAV (Fig. 5A, G), followed by culturing for an additional 10–12 days for subsequent experiments. Transfected cells were plated onto poly-d-lysine-coated glass coverslips for imaging.

*Preparation of brain slice*

To obtain recordings from acute brain slices, the mice were deeply anaesthetised with isoflurane and decapitated. Coronal brain slices (200 μm thick) containing the OFC were prepared using a vibratome (VT1000S; Leica, Wetzlar, Germany) filled with ice-cold cutting solution (120 mM NMDG-Cl, 2.5 mM KCl, 26 mM NaHCO_3_, 1.25 mM NaH_2_PO_4_, 0.5 mM CaCl_2_, 7 mM MgCl2, 15 mM d-glucose, and 1.3 mM ascorbic acid; pH 7.2). Slices were incubated in oxygenated ACSF (124 mM NaCl, 3 mM KCl, 26 mM NaHCO_3_, 1 mM NaH_2_PO_4_, 2.4 mM CaCl_2_, 1.2 mM MgCl_2_, and 10 mM d-glucose; pH 7.3) at 32°C for ≥ 1 h. After recovery, the individual slices were transferred to a recording chamber with continuous perfusion of oxygenated ACSF. ACSF was heated to maintain the recording chamber at 27°C ± 1°C. Recordings were performed within 4 h of recovery.

*Statistical test*

Between-group differences were analysed using a two-tailed Student’s *t*-test or unpaired *t*-test with Welch’s correction. Differences within each mouse were analysed using a two-tailed paired *t*-test. Among-group differences were analysed using one-way analysis of variance (ANOVA) with post-hoc Sidak’s multiple comparison test or two-way ANOVA with post-hoc Sidak’s multiple comparison test. A two-way ANOVA for repeated measures, followed by Tukey’s multiple comparisons tests, was used to analyse the results of the non-crossover design. A three-way ANOVA for repeated measures, followed by Holm–Sidak’s multiple comparisons tests, was used to analyse the results of lever presses in the devaluation test.
